# Exploring the Association Between Social Determinants of Health and Telehealth Utilization for Attention-Deficit/Hyperactivity Disorder Among Adults Using Machine Learning: A Cross-Sectional Study

**DOI:** 10.3390/healthcare14172709

**Published:** 2026-08-25

**Authors:** Weijian Qin, Yunshu Yang, Shiqin Tong, Dongze Li, Hang Liu, Zongbo Li, Hawking Yam, Jin Huang, Jose Florez-Arango

**Affiliations:** 1 Department of Population Health Science, Weill Cornell Medicine, New York, NY 10065, USA; weq4001@alumni.weill.cornell.edu (W.Q.); yang9291@umn.edu (Y.Y.);; 2 Division of Health Policy and Management, University of Minnesota, Minneapolis, MN 55455, USA; 3 School of Social Work, Columbia University, New York, NY 10027, USA; 4 Department of Economics, University of Southern California, Los Angeles, CA 90089, USA; 5 School of Public Health, Stanford University, Stanford, CA 94305, USA; 6 Center for Biomedical Informatics & Biostatistics, University of Arizona, Tucson, AZ 85721, USA

**Keywords:** telehealth, attention-deficit/hyperactivity disorder (ADHD), social determinants of health (SDOH), machine learning (ML), health equity, United States (U.S.), rapid surveys system (RSS)

## Abstract

Background: Attention-Deficit/Hyperactivity Disorder (ADHD) affects an estimated 6% of adults in the United States and contributes to a significant economic burden. Telehealth has emerged as a vital tool in the management of ADHD, offering improved access to care, especially for individuals in underserved communities. Despite its growing role, there remain critical gaps in understanding how social determinants of health (SDOH) are associated with disparities in telehealth utilization for ADHD treatment. Objectives and Methods: This study analyzed data from the National Center for Health Statistics (NCHS) Rapid Surveys System (RSS) Round 2: ADHD (October–November 2023), a nationally fielded survey of U.S. adults. Respondents were classified into three groups: never diagnosed, previously diagnosed, and currently diagnosed with ADHD. The study aimed to (1) compare the distribution of SDOH across ADHD status groups and the general adult population to identify factors associated with ADHD diagnosis; (2) assess the homogeneity of SDOH distributions across ADHD groups; (3) evaluate telehealth utilization among adults currently diagnosed with ADHD; and (4) examine the relationship between SDOH and telehealth use for ADHD treatment. Multivariable logistic regression (MVLR) served as a benchmark model, while machine learning (ML) models—including regularized linear regression, support vector machine (SVM), random forest (RF), LightGBM, multilayer perceptron (MLP), and Few-Shot Learning (FSL)—were trained to identify key predictors. Results: A total of 7009 survey responses were analyzed: 124 had a past diagnosis, 444 were currently diagnosed, and the remainder had never been diagnosed with ADHD, corresponding to a current ADHD prevalence of 6.3%. Adults with current ADHD were more likely to be male, single, younger, white, non-homeowners, and frequent users of online health resources. They also reported lower education, income, and financial security. About 70% used telehealth for counseling and prescriptions; insurance covered telehealth visits for 82.32% of users, yet 38.76% reported no coverage of ADHD-related diagnostic or treatment costs. Nineteen SDOH elements across four domains—demographic, socioeconomic, neighborhood/built environment, and healthcare access—were identified as predictors. ML models outperformed MVLR, with SVM and FSL achieving the highest F1 (both 0.63), and FSL the highest recall (0.69). Age, race, marital status, difficulty paying bills, home ownership, education, and household size were the most consistently important variables. Limitations: This study is limited by a cross-sectional design, reliance on self-reported ADHD diagnoses, and a lack of genetic or family-history measures. Additionally, the omission of complex sampling weights limits the national representativeness of these findings. Finally, the small effective sample size poses risks of model overfitting, and the generalizability of the models could not be externally validated due to the unavailability of comparable independent datasets. Conclusions: Despite widespread internet access, disparities in telehealth use for ADHD persist. Among 19 SDOH predictors, age (aOR = 0.56), difficulty paying medical bills (aOR = 2.52), and race (aOR = 1.37) were significantly associated with telehealth use, and all ML models outperformed the MVLR benchmark, though bootstrap CIs overlapped. Future research should incorporate inclusive data collection and stratified modeling to better represent disadvantaged populations and inform equitable access strategies.

## 1. Introduction

Attention-Deficit/Hyperactivity Disorder (ADHD), a condition marked by symptoms such as inattention, hyperactivity, and impulsivity [1], affects 2–5% of adults globally, and approximately 6% of U.S. adults [2,3]. It is the third most prevalent U.S. mental health condition, following anxiety and depression [4,5]. Over 70% of adults with ADHD have comorbidities, including depression (55%), anxiety (47%), substance use (41%), and bipolar disorder (35%) [6,7]. Beyond its core symptoms, ADHD in adults contributes to an estimated USD 122.8 billion annual economic burden in the U.S., driven largely by unemployment, productivity loss, and healthcare costs (USD 14,092 per adult in 2018) [8,9,10]. Treatment in the U.S. primarily relies on pharmacotherapy; stimulant medications remain the first-line, gold-standard option given their efficacy and favorable long-term safety profile often combined with cognitive behavioral therapy (CBT) [11,12,13].

The expansion of digital technologies has enabled remote delivery of mental health services, including diagnosis, therapy, and medication management [14]. A systematic review of meta-analysis from 2010 to 2019 found telehealth to be as effective or superior to usual care across multiple disciplines, including neurology, psychiatry, and psychology [15]. COVID-19 has been causing huge disruptions in mental healthcare services worldwide and more healthcare services transit to virtual care [16], further underscoring its importance, as ADHD patients required new modes of care [17]. Telehealth supports ADHD care across initial consultations, diagnoses, prescriptions, follow-ups, and medication management, improving access and addressing healthcare disparities [18]. To address access barriers, the DEA temporarily suspended Ryan Haight Act restrictions, allowing providers to prescribe controlled substances like buprenorphine and Adderall, via telehealth without in-person visits and have now been extended until the end of 2025 [19]. Telehealth especially benefits underserved groups, including racial and ethnic minorities, low-income individuals, Medicaid/Medicare recipients, patients with chronic conditions, and rural residents with limited access to mental health services [20,21,22]. Yet, evidence on whether these benefits are realized in practice is mixed, as the literature reviewed in Section 2 shows.

The uneven distribution of telehealth benefits across populations suggests that access depends on more than technology availability. Social determinants of health (SDOH), including economic stability, education access and quality, healthcare access and quality, neighborhood and built environment, and social and community context that shape health outcomes, are closely linked to telehealth use and ADHD diagnosis through complex biological, psychological, and social pathways [23,24,25,26]. In this study, we organize SDOH into four domains: demographic characteristics, socioeconomic status, neighborhood and built environment, and healthcare access and quality that map onto this established framework. Despite growing recognition that SDOH jointly shape health-seeking behavior, no study has jointly examined these domains as predictors of telehealth utilization among adults with current ADHD, nor compared conventional and machine learning (ML) approaches for this outcome, as detailed in Section 2. Understanding these relationships is critical for designing targeted interventions to promote health equity.

Traditional statistical methods such as multivariable logistic regression (MVLR) remain the standard for estimating adjusted associations and offer interpretable, directly reportable effect estimates, but they typically assume additive, linear relationships and require interaction terms to be specified in advance, an assumption that is difficult to satisfy when the relevant interactions among numerous correlated SDOH are not known ahead of time [27,28]. ML is well suited to exactly this challenge: it can flexibly model non-linear relationships and higher-order interactions among correlated SDOH variables without requiring them to be pre-specified, and several ML algorithms also produce built-in variable-importance rankings that support systematic comparison across predictors [29]. For a population as heterogeneous as adults with ADHD, who vary widely in socioeconomic circumstances, insurance status, and household structure, this flexibility may allow ML to explore which combinations of SDOH most strongly shape telehealth use, while MVLR continues to serve as an interpretable benchmark against which ML gains can be judged. Moreover, ML is increasingly used in SDOH research for prediction, causal inference, data curation, and algorithmic fairness [28], with worked examples reviewed in Section 2.

This study makes three contributions: characterizing SDOH across four domains for adults with current, remitted, and no ADHD diagnosis; quantifying telehealth utilization and insurance coverage for ADHD-related diagnosis, counseling, and prescribing among adults with current ADHD; and benchmarking seven ML algorithms against MVLR to identify which SDOH domains most consistently predict telehealth use, with affordability, coverage, and household resources emerging as the most robust. The remainder of the article proceeds as follows: Section 2 reviews related work; Section 3 describes the data source, measures, and analytic methods; Section 4 presents results; Section 5 discusses the findings and their implications; Section 6 presents limitations, and Section 7 concludes the findings.

## 2. Related Works

### 2.1. Telehealth Utilization Among Adults with ADHD

The most directly relevant evidence on ADHD-related telehealth use comes from the National Center for Health Statistics’ own analysis of the Rapid Surveys System Round 2 data used in the present study: Staley et al. reported that roughly half of U.S. adults with a current ADHD diagnosis have used telehealth for ADHD care and that stimulant-prescription fulfillment problems are widespread [3]. That report, however, is descriptive: it presents national prevalence estimates without examining which patient-level social or economic characteristics distinguish telehealth users from non-users, and it does not compare adults with current ADHD to those never diagnosed or in remission. Beyond this source study, the ADHD-telehealth literature consists largely of narrative and policy-oriented work on the effectiveness of telehealth for mental healthcare generally and on the regulatory flexibilities that expanded prescribing access, both discussed in the Introduction [15,16,18,19,20], and on the populations telehealth is presumed to benefit [21,22,23], rather than analyses that directly model utilization as an outcome.

### 2.2. The Role of SDOH in Telehealth Access and ADHD Care

A separate body of work has examined SDOH as drivers of telehealth access, largely outside the ADHD context. Vakkalanka et al.’s systematic review found that telehealth access disparities for mental health and substance use disorders widened, rather than narrowed, during the COVID-19 pandemic, particularly among rural, older, and Black/African American populations [26]. Other studies already cited later in this manuscript have linked broadband access, rurality, and socioeconomic disadvantage to lower telehealth uptake more broadly [30,31], and have documented persistent gaps in reimbursement and video-visit access among underserved patients [32,33,34]. Parallel work outside telehealth has shown that explicitly incorporating SDOH into clinical prediction models, rather than adjusting only for basic demographics, can materially change model performance and expose disparities that would otherwise be missed, for example, in orthopedic outcome prediction [35] and in-hospital mortality prediction for heart failure [36]. None of these studies, however, jointly examine SDOH and telehealth use among adults specifically diagnosed with ADHD, leaving open how the domains that matter for telehealth access, generally affordability, insurance, and household resources, operate in this particular clinical population.

### 2.3. ML Applications in Telehealth or ADHD Research

ML has been applied to telehealth and to ADHD as separate problems, but rarely to both together. On the telehealth side, Cengil et al. used random forest, XGBoost, and deep neural network models on a national outpatient dataset to predict telehealth resource use and found that Social Vulnerability Index household vulnerability was among the strongest predictors of use patterns [37]; Chen et al. similarly showed that a decision-tree model using only age, gender, race, and address-level SDOH could meaningfully risk-stratify healthcare utilization more broadly [38]. On the ADHD side, ML has mainly targeted diagnosis rather than service use: Chen et al. developed a hybrid machine learning and knowledge-based algorithm that raised adult ADHD diagnostic accuracy above 90% in an NHS clinical trial [39], and a recent review found that deep-learning models for early ADHD detection rarely incorporate comprehensive SDOH, limiting their ability to address diagnostic and access disparities [40]. To our knowledge, no prior study has applied ML to predict telehealth utilization specifically among adults with ADHD using a multi-domain SDOH feature set, or benchmarked such models against conventional regression for this outcome.

### 2.4. Positioning of the Present Study

Taken together, this literature leaves a gap at the intersection of the three streams above: the descriptive ADHD-telehealth literature does not model predictors, the SDOH-telehealth literature is not ADHD-specific, and ML has been applied to telehealth resource use and to ADHD diagnosis separately but not to telehealth utilization among adults with ADHD. The present study is positioned to close this gap; its specific contributions and hypotheses are stated at the end of the Introduction.

## 3. Materials and Methods

This study used de-identified, publicly available NCHS data to examine the association between SDOH and telehealth utilization for ADHD treatment among U.S. adults with ADHD using ML to identify key predictors, and explore complex patterns among predictors, adjusted for confounders [41,42,43]. Institutional Review Board (IRB) approval was not required because the data are publicly available and de-identified. The study followed STROBE guidelines for cross-sectional reporting [44].

### 3.1. Measures

#### 3.1.1. Study Population by Diagnosis Status

Participants were classified into three groups based on their responses to two survey questions:

(1) Undiagnosed: Adults who answered “no” to “Have you ever been diagnosed with ADHD by a doctor or other health professional?”
(2) Remitted ADHD: Adults who answered “yes” to the first question but “no” to “Do you currently have ADHD?”
(3) Current ADHD: Adults who answered “yes” to both questions. Separating remitted from current ADHD is clinically meaningful: individuals who meet remission criteria may still exhibit functional limitations and health-service needs, whereas those with current ADHD reflect persistent symptoms that typically require ongoing intervention. This grouping is supported by longitudinal and functional–outcome evidence showing these distinct adult outcomes [42,43].

#### 3.1.2. Social Determinants of Health

Potential SDOH include (1) demographic factors (age, gender, race/ethnicity, marital status), (2) socio-economic status (SES) (education, household size, income, poverty, employment, homeownership, and difficulties or concerns about paying medical or housing costs), (3) neighborhood and built environment (region, metropolitan status, and transportation barriers), and (4) healthcare access and quality (insurance coverage, having a usual source of care, and the type of care setting).

#### 3.1.3. Utilization of Telehealth Services for ADHD Patients

For adults currently diagnosed with ADHD, we assessed the utilization of telehealth services, which was defined based on responses to nine questions: They were first asked about receiving telehealth services for ADHD. If they answered “yes,” they were asked whether they had used telehealth for an initial ADHD visit, diagnosis (telehealth, in-person, or both), counseling or therapy, and obtaining ADHD medication since March 2020. Then they were asked whether health insurance covered any costs for their ADHD diagnosis or treatment in the past 12 months. Follow-up questions varied based on prior responses. Those using telehealth for an initial visit, prescriptions, or counseling were asked about insurance coverage for telehealth ADHD visits since March 2020. Participants who used telehealth for ADHD prescriptions were asked if they planned another telehealth visit for a prescription in the next three months, while those using telehealth for counseling or therapy were asked about plans for future sessions. The complete survey logic, including participant classification and telehealth assessment questions, is presented in Figure S1.

#### 3.1.4. Potential Confounders

Confounding variables include “internet accessibility” and “using internet to communicate with a doctor”, assessed by responding to two questions: “Do you have access to the Internet?” and if so, “In the last 12 months, have you used the Internet to communicate with a doctor or doctor’s office?” More details on the questionnaires are available at the CDC website [45]. These two variables were ultimately excluded from the regression and ML models owing to near-complete collinearity with the outcome.

### 3.2. Statistical Analysis

**Figure 1:**
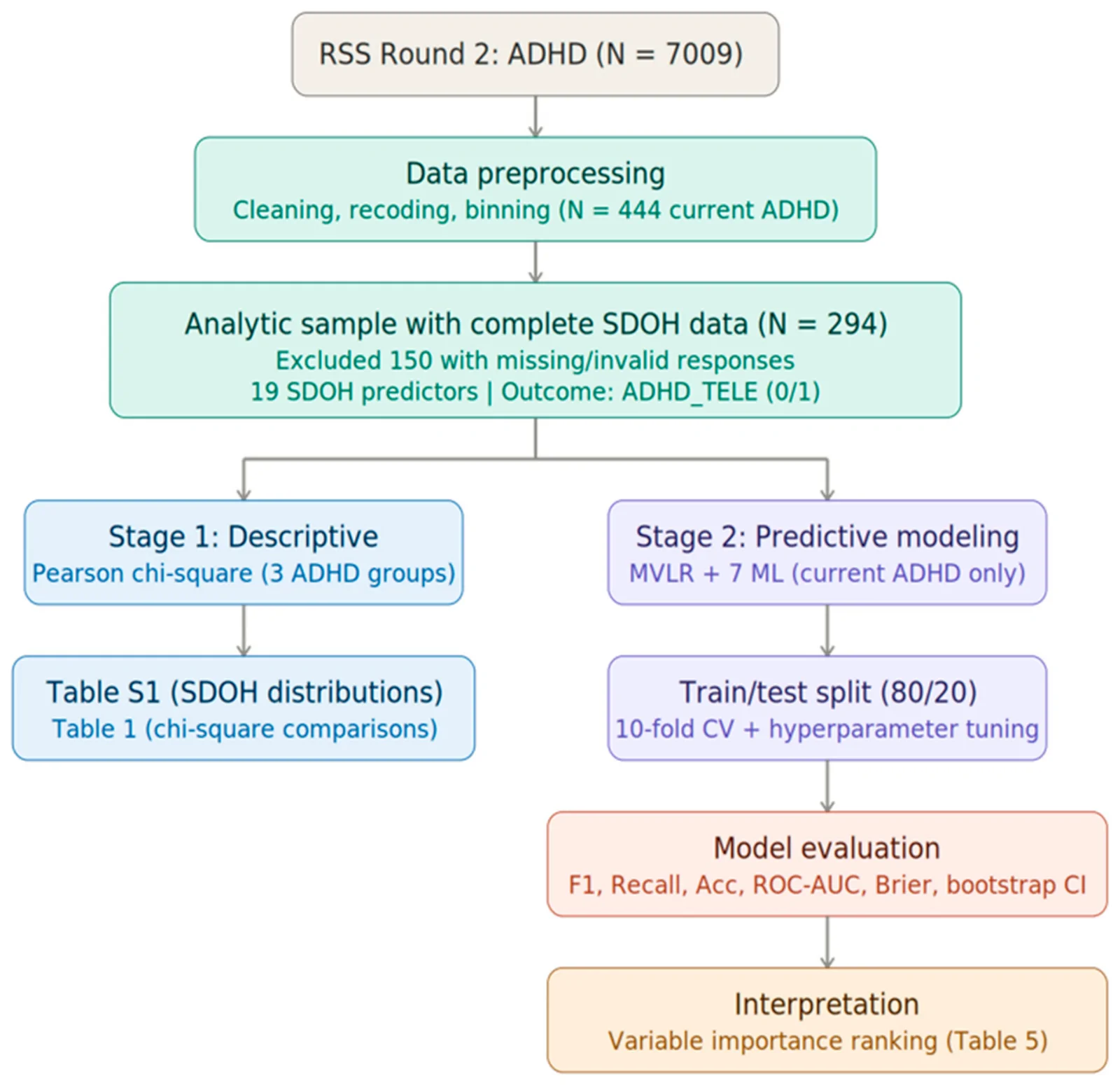
Study design and analysis overview. ADHD: Attention Deficit Hyperactivity Disorder, SDOH: Social Determinants of Health.

The analysis proceeded in two stages. First, descriptive statistics and Pearson chi-square tests compared SDOH distributions across all three ADHD diagnosis groups to characterize sociodemographic differences by diagnosis status. Second, MVLR and ML models were applied to the primary analytic population—adults currently diagnosed with ADHD with complete SDOH data (*N* = 294)—to identify variables associated with telehealth utilization. The overall study design is illustrated in Figure 1 (Study design and analysis overview). The dataset was initially cleaned to address inconsistencies, particularly within the variables “Have you ever received any telehealth services for ADHD?” and “In the past 12 months, have you used the internet to communicate with a doctor or doctor’s office?”. Twenty-five conflicting responses were recoded. All invalid, missing value, or non-informative legitimate skip responses were excluded. Proportion analyses compared three ADHD status groups with the general population to identify SDOH associated with ADHD diagnosis status. Pearson chi-square tests of independence, including pairwise comparisons, were performed to assess differences in SDOH distributions among the three ADHD status groups, with chi-square values (*χ*^2^), statistical significance was set at *p* less than 0.05, and degrees of freedom (df) reported. To maintain methodological consistency, all analyses were conducted without survey sampling weights, as the ML models used in the second stage do not fully accommodate the complex survey design (weights, stratification, and clustering), and the primary aim was to examine associations rather than to produce nationally representative estimates.

Among adults currently diagnosed with ADHD, telehealth utilization was assessed. Percentages and the corresponding 95% confidence intervals (CIs) for telehealth utilization variables were calculated using the normal approximation method (Wald interval). Then, multicollinearity was assessed via variance inflation factors (VIF); MVLR was used as the benchmark ML model to estimate SDOH–telehealth associations for ADHD, reported as adjusted odds ratios (aORs) with 95% confidence intervals (CIs) and *p* values. To identify the key predictors for telehealth utilization among the current ADHD group through a complex data pattern, we trained seven prespecified ML algorithms, and selected by the past literature and dataset suitability, covering regularized regression, kernel-based, tree-based, neural network, and prototype-based approaches. Lasso/Elastic Net provide regularized, interpretable selection (handling correlated SDOH); SVM maximizes margins with good small-sample generalization and captures nonlinear boundaries; RF/LightGBM model nonlinearities and interactions with minimal tuning (LightGBM also handles missingness and class imbalance) and yield variable importance; a compact MLP learns higher-order patterns with dropout/weight decay and early stopping; and a Prototypical Network (FSL), which classifies by computing distances to class-level centroids in a learned embedding space rather than via softmax output, was included for its simpler inductive bias and reduced overfitting risk in moderate-sample settings [46]. The primary outcome variable was ADHD_TELE, a binary indicator of whether the respondent had ever received telehealth services for ADHD. Of the 441 adults currently diagnosed with ADHD who provided valid responses, 294 had complete data on all 19 SDOH predictors and were retained for the MVLR and ML analyses. Within this cohort, 136 participants (46.3%) reported telehealth use for ADHD and 158 (53.7%) did not, corresponding to a nearly balanced outcome distribution (1:1.16 ratio). We applied class_weight = “balanced” to Lasso, Elastic Net, SVM, random forest, and LightGBM to ensure robust performance across both classes. The primary source of missing data was health insurance coverage (*n* = 145, 33.0%), followed by type of usual care place (*n* = 40, 9.1%); the remaining variables had less than 2% missing. To assess potential selection bias from complete-case analysis, we compared the 294 included and 147 excluded participants on key demographic and SDOH variables using chi-square tests. The primary outcome (telehealth use) did not differ significantly between groups (46.3% vs. 44.2%, *p* = 0.685), nor did difficulty paying medical bills (*p* = 0.937), race (*p* = 0.073), or education (*p* = 0.185). However, excluded participants were significantly younger (27.4% vs. 9.9% aged 18–24, *p* < 0.001) and more often male (61.0% vs. 47.6%, *p* = 0.011), suggesting that complete-case analysis may slightly underrepresent younger male adults with ADHD. The absence of significant differences in the primary outcome and key predictors suggests that selection bias is unlikely to substantially alter the main findings. All 19 SDOH variables were entered into each model without further reduction. The dataset was randomly divided into training (80%) and test (20%) sets, and ten-fold cross-validation was conducted to mitigate overfitting and enhance model robustness. Tuning followed a two-stage approach—RandomizedSearchCV (10 iterations) followed by GridSearchCV for fine-tuning—conducted exclusively within the training folds using F1 as the scoring metric to prevent information leakage from the test set. Final hyperparameter values for each model are reported in Supplementary Table S2.

Model performance was evaluated using F1 score (harmonic mean of precision and recall), recall (proportion of true positives correctly identified), accuracy (proportion of correct predictions), ROC-AUC (area under the receiver operating characteristic curve), and Brier score (mean squared difference between predicted probabilities and observed outcomes). Variable importance was assessed using each algorithm’s native metric—coefficient magnitude for regularized regression and MVLR, Gini importance for random forest, split-based importance for LightGBM, and permutation importance for MLP and FSL.

All analyses were conducted using Python v 3.12. More details on public coding are available on GitHub [47].

## 4. Results

### 4.1. Data Descriptive Analysis

#### Participant Characteristics

Among 7009 adults, 6441 were never diagnosed, 124 had a past (remitted) diagnosis, and 444 were currently diagnosed with ADHD (current ADHD prevalence, 6.3%). Table S1 reports distributions for SDOH by ADHD-status group and overall. Age distributions differed across groups (*p* < 0.001): the overall sample skewed older (largest share aged 60–70 years), whereas adults with ADHD were disproportionately 25–44 years; the remitted group generally fell between the never-diagnosed and current-ADHD groups.

Never-diagnosed: By design, distributions closely tracked the overall sample; this group was older on average and showed higher levels of social and economic stability (higher educational attainment, home ownership, and insurance coverage; lower financial strain).

Remitted ADHD: Profiles were typically intermediate between the never-diagnosed and current-ADHD groups, with selective differences from the overall sample in marital and employment status, home ownership, financial strain, transportation reliability, and usual place/type of care.

Current ADHD: Relative to the overall sample, adults with current ADHD were younger and more often male and White, and differed on multiple socioeconomic indicators: lower educational attainment, lower income-to-poverty ratios, higher unemployment/being unable to work, greater financial strain (e.g., difficulty paying medical bills, worry about medical costs, inability to pay living expenses), and lower rates of home ownership and health-insurance coverage. In the access domain, lack of reliable transportation and differences in usual type of care were more common. Internet availability exceeded 98% in all groups, but reported internet use for doctor communication was highest among those with current ADHD.

### 4.2. Chi-Square Results of SDOH of Participants by ADHD Status Groups

The Chi-square tests examined the association between different SDOH and three ADHD status groups. The results of Table 1 indicate significant disparities based on demographic, socioeconomic, and healthcare access factors.

Age and marital status exhibited significant differences between the Never–Remitted and Never–Now groups but showed no significant differences in the Now–Remitted group (age: χ^2^ = 255.29, *p* < 0.001; marital status: χ^2^ = 119.90, *p* < 0.001 for Never–Now). This suggests that younger and unmarried individuals are more represented in the currently diagnosed group compared to the other two. Socioeconomic factors, including household income to poverty ratio, employment status, homeownership, and financial stress related to medical bills, concern about bill payment when ill, and inability to cover living expenses within 12 months, exhibited a similar pattern, with difficulty paying medical bills (χ^2^ = 191.70, *p* < 0.001), inability to pay living expenses (χ^2^ = 219.13, *p* < 0.001), and lack of reliable transportation (χ^2^ = 229.14, *p* < 0.001) showing the largest chi-square values among socioeconomic and access-related variables. Transportation access and healthcare access variables, such as whether they have a usual place to go for care and type of place for usual care, also shaped disparities between groups in utilization. In contrast, neighborhood-level factors such as metropolitan status and census region showed no significant differences across any group comparison.

These findings suggest that barriers to telehealth adoption for ADHD extend beyond technology availability, reflecting broader socioeconomic and healthcare inequities. Addressing these disparities is essential for ensuring equitable access to telehealth services for ADHD management.

### 4.3. ADHD-Related Telehealth Utilization Status

Table 2 summarizes telehealth utilization among adults with a current ADHD diagnosis. Overall, 45.58% had ever used telehealth services for ADHD, and 50.50% reported using telehealth for their first ADHD-related visit. Regarding diagnosis, 23.12% were diagnosed via telehealth, while the majority received their diagnosis in person (58.79%) or through a combination of in-person and telehealth visits (18.09%). Among those with ADHD, 70.50% had used telehealth for counseling or therapy and 68.34% for prescription services. Insurance paid ADHD-related diagnostic or treatment costs for 61.24% of adults with current ADHD, and 82.32% of those who used telehealth for a first visit, prescriptions, or counseling reported insurance payment for telehealth ADHD visits In terms of continuity, 67.24% planned another telehealth visit for ADHD prescriptions and 75.21% for counseling sessions in the next three months. These findings highlight the widespread role of telehealth in ADHD management, alongside remaining gaps in insurance coverage.

### 4.4. Multivariable Logistic Regression Analysis

To examine factors associated with telehealth utilization among adults with ADHD, we conducted the MVLR (Table 3). The model failed to converge when internet accessibility and using the internet to communicate with a doctor were included, due to high collinearity between these variables and the outcome. For comparability across models, these variables were excluded from the MVLR as well as from the subsequent ML models. Results showed that older adults had lower odds of telehealth use (aOR = 0.56, 95% CI [0.36, 0.87], *p* = 0.011). Difficulty paying medical bills was associated with higher odds of telehealth use (aOR = 2.52, 95% CI [1.16, 5.52], *p* = 0.020), and race was also significantly associated with telehealth utilization (aOR = 1.37, 95% CI [1.05, 1.79], *p* = 0.021). Other variables, including marital status, insurance coverage, and homeownership, showed positive associations with telehealth use but did not reach statistical significance at the 0.05 level.

While the MVLR provided useful insights, it may not fully capture complex or non-linear relationships among predictors and outcome. Given these findings, ML approaches could improve predictive performance and robustness.

### 4.5. ML Models Performance

#### 4.5.1. Overall Model Performance

**Figure 2:**
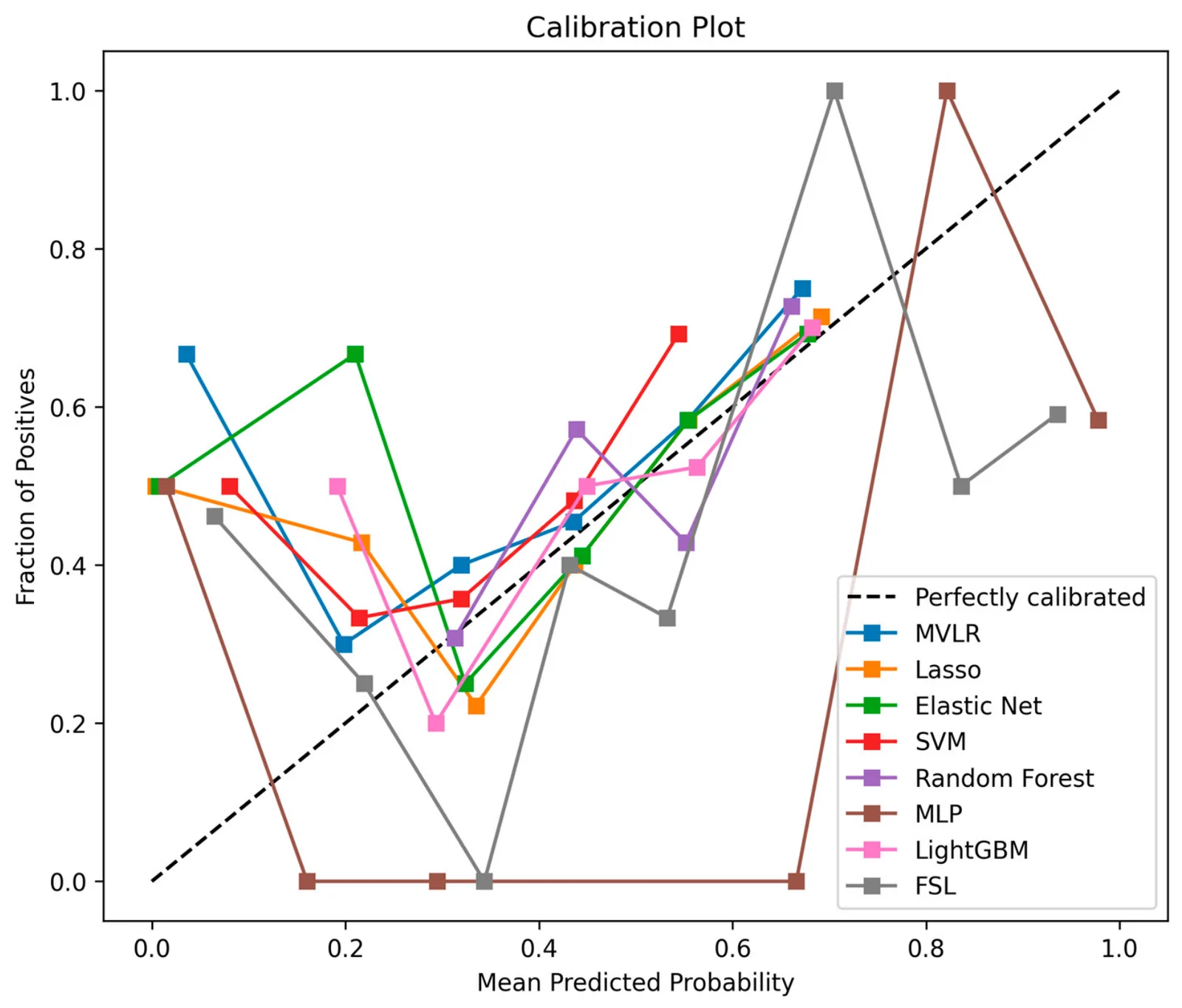
Calibration curves for all models.

Compared with the MVLR benchmark (F1 = 0.53; recall = 0.45; accuracy = 0.61), all ML models achieved higher F1 scores (0.56–0.63). SVM and FSL yielded the highest F1 (both 0.63), with FSL achieving the highest recall (0.69) and SVM the highest accuracy (0.66) (Table 4). Lasso (F1 = 0.62) and LightGBM (F1 = 0.60) also performed well. Bootstrap 95% confidence intervals overlapped across all models, indicating that performance differences were not statistically significant given the modest sample size. Model calibration varied across algorithms Figure 2 (Calibration curves for all models). Calibration curves were generated by binning predicted probabilities into deciles and computing observed telehealth use frequency within each bin, where points along the diagonal indicate well-calibrated predictions. Random forest and LightGBM tracked the diagonal closely (Brier = 0.235 and 0.242), while MLP systematically overestimated telehealth use probability (Brier = 0.394).

#### 4.5.2. Predictors Across Models

Within each algorithm, predictors were ranked by the model’s native importance metric (1 = most important). “Top-rank” was defined a priori as rank ≤ 10 of 19 predictors. Cross-model consistency was summarized by *(i)* the number of algorithms (of 8) in which a predictor was top-ranked and *(ii)* its median rank. Predictors meeting both thresholds—top-rank in ≥6/8 models and median rank ≤ 10—were classified as “consistently high-ranking”; those top-rank in 4–5 models were “often high-ranking”; ≤3 models were “model dependent.” By these criteria, seven predictors were consistently high-ranking—age, race, marital status, difficulty paying medical bills, home ownership, education level, and household size (median ranks ≈ 3–9). Health insurance coverage and usual place for care were often high-ranking (top-10 in 4–5 models). The remaining predictors were model dependent, tending to rank highly only in particular models (e.g., income in MLP; poverty ratio in random forest). As the heatmap (Figure S2) illustrates, variables such as metropolitan status and income showed high rankings only in specific algorithms (e.g., income in MLP; metropolitan status in FSL), contrasting with the cross-algorithm consistency of age and financial hardship. Overall, affordability/coverage and household resources emerged as the most stable correlates of telehealth use. Details of predictor rankings across all ML algorithms are presented in Table 5.

## 5. Discussion

Our findings show that adults currently diagnosed with ADHD were more likely to be male, White, and report unreliable transportation patterns consistent with prior research. (1) A 2025 report highlighted persistent gender, racial, and ethnic disparities in adult ADHD diagnosis, with White adults and men more likely to receive a diagnosis [48,49,50,51]. Additionally, 55.9% of respondents with current ADHD were aged 25–44, aligning with national prevalence estimates from CHADD [52], reaffirming that ADHD remains highly prevalent among young and middle-aged adults. (2) Regarding SES, participants with ADHD were more likely to have lower educational attainment, be unmarried, report lower income, be unemployed, and experience financial insecurity related to medical bills, housing, and emergencies. These findings reinforce prior evidence linking SES disadvantage to ADHD diagnosis and outcomes [53,54,55,56,57,58,59]. Moreover, Mendelian randomization studies suggest a bidirectional relationship: lower education and income levels increase ADHD risk, while ADHD exacerbates educational and employment challenges [60], embedding ADHD within broader public health and social structures. Interestingly, no significant difference was found in metropolitan residency between adults with ADHD and the general population, contrasting with prior research suggesting higher ADHD prevalence in urban areas [61]. Future research should further examine geographic patterns in the context of evolving telehealth access and urban migration trends. Together, these findings highlight the intersection of demographic, socioeconomic, and structural factors in ADHD diagnosis and care disparities.

Key predictors of telehealth utilization for ADHD varied across ML models; however, seven factors consistently ranked among the most influential factors. **(1) Internet access:** We excluded internet accessibility and use for provider communication from the models due to multicollinearity, yet reliance on internet-based data collection in RSS-2 and the modest completion rate (37.2%) suggest underrepresentation of digitally marginalized groups. Prior studies, however, have shown that older adults, Black individuals, and those with lower education levels are less likely to use telehealth [30], largely reflecting persistent infrastructural disparities [31]. The frequent importance of metropolitan residence was model dependent in the present analysis, though prior evidence suggests that rural populations face ongoing digital and provider shortages, reinforcing urban–rural gaps in telehealth access [55]. **(2) SES:** Several SES predictors emerged as consistently important predictors. Financial hardship, including difficulty paying medical bills, and lack of homeownership particularly associated with reduced access. Higher educational attainment may also be associated with understanding of the ADHD diagnostic process, proactive help-seeking, and adherence to treatment [32]. Larger household sizes may limit access to telehealth by requiring family members to share devices and physical space, which can compromise both availability and privacy [33]. Although employment status did not consistently rank among the top predictors across ML models, prior literature suggests it may affect scheduling flexibility for telehealth appointments, particularly for individuals with ADHD facing workplace challenges [34]. **(3) Health insurance coverage:** Coverage was a central correlate; inadequate insurance restricts assessment, prescriptions, and ongoing treatment, with disparities magnified in telehealth contexts [33,34]. **(4) Demographic patterns:** Age, race, and marital status also emerged as consistently high-ranking. Middle-aged and older adults are less likely to use telehealth than younger adults [3]. Significant racial/ethnic disparities exist in ADHD diagnosis rates, and some ethnic groups may face structural barriers to accessing remote ADHD services [62]. Among children covered by public insurance, the COVID-19 pandemic increased telemental health use overall, but the rise was significantly greater for white children, exacerbating racial/ethnic disparities. This study examined ages 3–17 and excluded adults with ADHD, leaving a gap in research on race and ethnic disparities in the use of telemental healthcare for adults with ADHD [63]. Patients who are married or have a partner may have more emotional and time support and thus be more inclined to use telehealth services for ADHD [51].

Selecting appropriate ML models helps elucidate these interrelationships. All ML models outperformed the MVLR benchmark, indicating better capture of the complexity of telehealth use. Among them, SVM demonstrated the most balanced performance, likely due to its ability to model nonlinear boundaries and heterogeneous subgroups, whereas MVLR could not accommodate such conditional dependencies or interaction effects. These contrasts reflect differences in modeling assumptions. Nonlinear methods such as SVM and random forest can accommodate threshold effects and synergistic influences (e.g., financial strain interacting with lack of insurance and large household size), which can be advantageous in small, heterogeneous samples. By contrast, linear models like MVLR do not capture these complexities without prespecified interaction terms. Tree-based methods, including LightGBM, are theoretically well suited for capturing variable interactions and ranking predictor importance, but their reliance on larger and more balanced datasets may explain their relatively weaker performance in this study.

From a clinical perspective, the modest absolute improvement of ML over MVLR (F1 from 0.53 to 0.63) suggests that the primary value of this analysis is not in prediction accuracy itself, but in identifying actionable SDOH correlates. For instance, difficulty paying medical bills (aOR = 2.52) indicates that adults reporting financial hardship were more than twice as likely to use telehealth, while older adults were approximately half as likely (aOR = 0.56). Screening for difficulty paying medical bills during clinical intake could help identify patients who may benefit from telehealth referral or insurance navigation support, though this study demonstrates higher utilization rates rather than improved outcomes among this group. Whether telehealth referral based on financial hardship improves clinical outcomes should be evaluated through prospective studies.

These gains come with a fairness caveat: several top predictors—race, insurance coverage, and homeownership—are themselves markers of structural inequity, so models trained on this observational data risk reproducing existing disparities rather than identifying modifiable barriers. A model that ranks insurance or homeownership highly could, if used for outreach, systematically deprioritize already disadvantaged subgroups. Given the modest current analytical sample (*n* = 294), we did not test subgroup-stratified performance or calibration. Future studies with larger samples should evaluate subgroup-stratified ROC curves and calibration slopes across race/ethnicity and insurance status, and implement fairness-aware metrics (e.g., equalized odds) during model training.

Given the modest predictive performance (ROC-AUC 0.60–0.65), this analysis is best positioned for hypothesis generation and SDOH correlate identification rather than individual-level prediction or operational deployment. The consistent importance of age, financial hardship, and race across algorithms suggests these are robust correlates warranting further investigation through mechanistic and longitudinal studies.

## 6. Limitations

Several limitations warrant consideration. First, the cross-sectional design captures a single time point and precludes assessment of temporal trends or causal inference. The modest sample size further limits generalizability and statistical power. In particular, variable importance rankings may be unstable given the sample-to-predictor ratio, and some findings may not replicate in larger, independent datasets. Relatedly, the bootstrap confidence intervals overlapped across all ML models and with the MVLR benchmark (Table 4), indicating that observed performance differences did not reach statistical significance and should be interpreted with caution. Second, although ADHD is highly heritable (70–80%) [64], the dataset lacked genetic or family-history measures, limiting adjustment for hereditary influences on diagnosis and telehealth use. Third, ADHD diagnoses were based on self-reported data, which may be subject to cognitive and language biases, misreporting, or invalid self-diagnoses without medical verification. Fourth, the analysis relies on unweighted survey data, and Pearson chi-square tests were used rather than survey design-adjusted methods (e.g., Rao-Scott). This may underestimate standard errors and inflate statistical significance, although the overall pattern of associations is expected to remain consistent. Variable importance rankings may also shift under weighted analyses, and findings should be interpreted as describing survey respondents rather than the U.S. adult ADHD population. Finally, due to the unavailability of alternative independent datasets containing comparable variables, external validation could not be conducted to further verify the generalizability of the trained machine learning models.

## 7. Conclusions

This study suggests that SDOH play an important role in telehealth utilization among adults with ADHD. Among 19 SDOH predictors, age (aOR = 0.56), difficulty paying medical bills (aOR = 2.52), and race (aOR = 1.37) were significantly associated with telehealth use in the MVLR, and these variables—along with household size and education level—were consistently high-ranking across seven ML algorithms. Although all ML models achieved numerically higher F1 scores than MVLR, bootstrap confidence intervals overlapped, indicating no statistically significant performance differences.

To our knowledge, this is the first study to examine the relationship between SDOH and telehealth utilization specifically among U.S. adults with ADHD using multiple ML approaches on a nationally fielded survey. Future research should use longitudinal designs to assess temporal trends, larger and more diverse samples to improve model stability, and fairness-aware metrics to ensure predictive models do not reproduce existing disparities. From a policy perspective, strengthening insurance protection, enhancing health literacy, and providing culturally competent care may help address structural barriers and promote more equitable use of telehealth in mental health across diverse populations.

## Figures and Tables

**Table 1:** Pairwise chi-square comparisons of SDOH distributions across ADHD status groups.

Factors	Never → Remit	Never → Remit	Never → Now	Never → Now	Now → Remit	Now → Remit	df
	*Chi2*	*p*	*Chi2*	*p*	*Chi2*	*p*	
***Demographic Factors***							
Age	68.2888	0.0000 *	255.2942	0.0000 *	3.7788	0.2864	3
Gender	3.7936	0.0515	6.1238	0.0133 *	0.2470	0.6192	1
Race	8.4651	0.0373 *	9.5592	0.0227 *	13.4271	0.0038 *	3
Marital Status	20.3232	0.0001 *	119.8989	0.0000 *	2.5930	0.4587	3
***Socioeconomic Factors***							
Education Level	3.4017	0.1825	10.3909	0.0055 *	0.0481	0.9762	2
Household Size	10.0163	0.0748	47.0956	0.0000 *	2.0290	0.8451	5
Household Income	8.5340	0.0739	1.5700	0.8142	4.1303	0.3887	4
Household Income to Poverty Ratio	23.8965	0.0024 *	58.3453	0.0000 *	10.2288	0.2493	8
Employment Status	20.8817	0.0001 *	115.0253	0.0000 *	4.1098	0.2499	3
Home Ownership	30.6147	0.0000 *	79.0438	0.0000 *	0.7491	0.6876	2
Difficulty Paying Medical Bills	27.9123	0.0000 *	191.7040	0.0000 *	2.0711	0.1501	1
Worry About Paying Medical Bills if Sick	10.1954	0.0061 *	122.3804	0.0000 *	4.9897	0.0825	2
Unable to Pay Living Expenses	25.6546	0.0000 *	219.1294	0.0000 *	3.1282	0.0769	1
***Neighborhood and Built Environment***							
Metropolitan Status	0.0716	0.7890	1.2717	0.2595	0.5792	0.4466	1
Census Region	2.9780	0.3950	4.1657	0.2441	0.8611	0.8348	3
Lack of Reliable Transportation	61.7158	0.0000 *	229.1395	0.0000 *	0.0712	0.7896	1
***Healthcare Access and Quality***							
Health Insurance Coverage	5.2966	0.0709	24.9783	0.0000 *	0.3604	0.8351	2
Whether Has a Usual Place for Care	9.1776	0.0102 *	4.1153	0.1278	4.8606	0.0880	2
Type of Usual Care Place	27.1314	0.0001 *	22.6522	0.0004 *	5.4265	0.3661	5
***Internet Usage***							
Internet Accessibility	0.5100	0.4751	2.1734	0.1404	0.0000	1.0000	1
Internet Use for Doctor Communication	0.0000	1.0000	53.6000	0.0000 *	16.0157	0.0000 *	1

Note: * Indicates statistical significance (*p* < 0.05).

**Table 2:** Telehealth utilization for ADHD among adults with current diagnosis.

Variables	Category	Unweighted No.	Currently Have ADHD (%)	Unweighted ^†^ % (95% CI)
**Ever received telehealth services for ADHD**	No	240	54.42	[0.4977, 0.5907]
	Yes	201	45.58	[0.4093, 0.5023]
Universal: ADHD NOW = 1	Whole	441		
**Ever used telehealth for first-time visit for ADHD**	No	99	49.5	[0.4257, 0.5643]
	Yes	101	50.5	[0.4357, 0.5743]
Universal: ADHD TELE = 1	Whole	200		
**Diagnosed ADHD during telehealth, in-person, or both**	Telehealth visits	46	23.12	[0.1726, 0.2897]
	In-person visits	117	58.79	[0.5196, 0.6563]
	Both telehealth and in-person visits	36	18.09	[0.1274, 0.2344]
Universal: ADHD TELE = 1	Whole	199		
**Ever used telehealth for counseling/therapy for ADHD**	No	59	29.5	[0.2318, 0.3582]
	Yes	141	70.5	[0.6418, 0.7682]
Universal: ADHD TELE = 1	Whole	200		
**Ever used telehealth for ADHD prescription**	No	63	31.66	[0.252, 0.3812]
	Yes	136	68.34	[0.6188, 0.748]
Universal: ADHD TELE = 1	Whole	199		
**Insurance paid diagnostic/treatment costs for ADHD**	No	169	38.76	[0.3419, 0.4333]
	Yes	267	61.24	[0.5667, 0.6581]
Universal: ADHD NOW = 1	Whole	436		
**Insurance paid costs for telehealth ADHD visits**	No	32	17.68	[0.1212, 0.2324]
	Yes	149	82.32	[0.7676, 0.8788]
Universal: ADHD FIRST = 1 OR ADHD TELERX = 1 OR ADHD COUNSEL = 1	Whole	181		
**Plan another telehealth visit for ADHD prescription (next 3 months)**	No	38	32.76	[0.2422, 0.413]
	Yes	78	67.24	[0.587, 0.7578]
Universal: ADHD TELERX = 1	Whole	116		
**Plan another telehealth visit for ADHD counseling/therapy (next 3 months)**	No	29	24.79	[0.1696, 0.3261]
	Yes	88	75.21	[0.6739, 0.8304]
Universal: ADHD COUNSEL = 1	Whole	117		

Note: ^†^ Unweighted proportions calculated among respondents with valid responses; 95% CI calculated using the Wald method. Follow-up questions were asked only to respondents who answered “Yes” to prior items; sample sizes therefore decrease across subsequent questions. See Figure S1 for the complete survey logic. Bold rows indicate survey items; the "Universal" row beneath each item specifies the subpopulation to which the item was administered.

**Table 3:** Multivariable logistic regression results for predictors of telehealth utilization among adults with ADHD.

Variable	Adjusted OR	95% CI	*p*
Age	0.56	[0.36, 0.87]	0.011 *
Difficult to Pay Medical Bills	2.52	[1.16, 5.52]	0.020 *
Race	1.37	[1.05, 1.79]	0.021 *
Household Size	0.81	[0.61, 1.05]	0.115
Education Level	1.41	[0.91, 2.19]	0.126
Health Insurance Coverage	2.21	[0.68, 7.16]	0.187
Marital Status	1.14	[0.91, 1.45]	0.26
Usual Place For Care	1.11	[0.81, 1.52]	0.53
Poverty Ratio	0.93	[0.71, 1.20]	0.564
Income	1.27	[0.51, 3.16]	0.61
Lack of Reliable Transportation	1.09	[0.78, 1.53]	0.618
Employment Status	1.07	[0.80, 1.42]	0.662
Metropolitan Status	0.83	[0.36, 1.91]	0.664
Gender	1.14	[0.63, 2.04]	0.666
Home Ownership	1.1	[0.59, 2.05]	0.769
Worry about Medical Bills	1.05	[0.71, 1.55]	0.792
Census Region	0.97	[0.72, 1.31]	0.843
Type of Place for Usual Care	0.99	[0.89, 1.11]	0.866
Unable to Pay Living Expense	1.05	[0.57, 1.96]	0.87

Note: * Indicates statistical significance (*p* < 0.05).

**Table 4:** Machine learning model performance.

ML Algorithm	F1 (95% CI)	Recall	Accuracy	ROC-AUC (95% CI)	Brier
SVM	0.63 [0.47, 0.77]	0.5862	0.661	0.65 [0.51, 0.79]	0.247
FSL	0.63 [0.48, 0.75]	0.6897	0.5932	0.60 [0.46, 0.74]	0.316
Lasso	0.62 [0.45, 0.75]	0.5862	0.6441	0.63 [0.48, 0.77]	0.247
LightGBM	0.60 [0.45, 0.74]	0.6207	0.5932	0.62 [0.48, 0.75]	0.242
Elastic Net	0.59 [0.42, 0.74]	0.5517	0.6271	0.63 [0.49, 0.78]	0.245
MLP	0.56 [0.39, 0.70]	0.5517	0.5763	0.59 [0.43, 0.73]	0.394
Random Forest	0.56 [0.41, 0.69]	0.5862	0.5424	0.64 [0.51, 0.78]	0.235
MVLR (Benchmark)	0.53 [0.35, 0.69]	0.4483	0.6102	0.63 [0.48, 0.77]	0.253

**Table 5:** Comparative rankings of predictor importance across ML algorithms.

Variable	Median Rank	Random Forest	SVM	Elastic Net Reg	Lasso Reg	FSL	MLP	LightGBM	MVLR
**Consistently High-Ranking** (Top-10 in ≥6 of 8 algorithms and median rank *≤* 10)									
Age	2	1	2	2	2	7	8	1	3
Difficult to Pay Medical Bills	2	5	1	1	1	14	16	3	1
Race	4	6	7	3	3	1	2	4	5
Household Size	5	4	6	5	5	4	10	2	6
Marital Status	6	2	3	6	7	2	19	5	9
Education Level	6	8	5	4	4	11	7	6	4
Home Ownership	9	7	13	8	9	10	3	7	12
**Often High-Ranking** (Top-10 in 4–5 of 8 algorithms)									
Health Insurance Coverage	8	19	4	7	6	8	13	18	2
Usual Place For Care	10	10	12	9	8	18	5	8	11
**Model Dependent** (Top-10 in 3 of 8 algorithms)									
Poverty Ratio	11	3	9	17	15	6	11	11	16
Lack of Reliable Transportation	12	12	8	12	13	12	9	10	13
Metropolitan Status	12	18	17	13	11	5	4	17	8
Gender	14	14	15	10	12	16	18	14	10
Income	14	13	16	15	18	17	1	12	7
Unable to Pay Living Expense	15	17	18	14	14	15	12	19	14
Employment Status	15	15	14	11	10	13	15	16	15
Worry about Medical Bills	15	9	10	18	19	3	17	13	17
Census Region	16	11	19	16	16	9	14	15	18
Type of Place for Usual Care	17	16	11	19	17	19	6	9	19

## Data Availability

The data analyzed in this study are publicly available from the National Center for Health Statistics (NCHS) Rapid Surveys System (RSS) Round 2: ADHD, accessible at https://www.cdc.gov/nchs/rss/round2/ADHD.html (accessed on 12 August 2026).

## Supplementary Materials

The following supporting information can be downloaded at https://www.mdpi.com/article/10.3390/healthcare14172709/s1, Figure S1: Utilization of Telehealth Services for ADHD Patients, Figure S2: Variable Importance Ranking Across ML Algorithms, Table S1: The Distributions for SDOH by ADHD-Status Group, Table S2: Hyperparameters details.

## Abbreviations

The following abbreviations are used in this manuscript:

ADHD	Attention-Deficit/Hyperactivity Disorder
HHS	Department of Health and Human Services
ML	Machine Learning
RSS	Rapid Surveys System
SDOH	Social Determinants of Health
aOR	Adjusted Odds Ratio
CBT	Cognitive Behavioral Therapy
CDC	Centers for Disease Control and Prevention
CHADD	Children and Adults with Attention-Deficit/Hyperactivity Disorder
CI	Confidence Interval
DEA	Drug Enforcement Administration
df	Degrees of Freedom
DL	Deep Learning
FSL	Few-Shot Learning
IRB	Institutional Review Board
LightGBM	Light Gradient Boosting Machine
MLP	Multilayer Perceptron
MVLR	Multivariable Logistic Regression
NCHS	National Center for Health Statistics
RF	Random Forest
SES	Socioeconomic Status
STROBE	Strengthening the Reporting of Observational Studies in Epidemiology
SVM	Support Vector Machine
VIF	Variance Inflation Factor

## References

[1] National Institute of Mental Health (2024). Attention-Deficit/Hyperactivity Disorder: What You Need to Know.

[2] Ayano G., Tsegay L., Gizachew Y., Necho M., Yohannes K., Abraha M., Demelash S., Anbesaw T., Alati R. (2023). Prevalence of attention deficit hyperactivity disorder in adults: Umbrella review of evidence generated across the globe. Psychiatry Res..

[3] Staley B.S., Robinson L.R., Claussen A.H., Katz S.M., Danielson M.L., Summers A.D., Farr S.L., Blumberg S.J., Tinker S.C. (2024). Attention-Deficit/Hyperactivity Disorder Diagnosis, Treatment, and Telehealth Use in Adults—National Center for Health Statistics Rapid Surveys System, United States, October–November 2023. MMWR-Morb. Mortal. Wkly. Rep..

[4] National Alliance on Mental Illness (2023). Mental Health by the Numbers. https://www.nami.org/about-mental-illness/mental-health-by-the-numbers/.

[5] National Academies of Sciences, Engineering, and Medicine, Health and Medicine Division, Board on Health Sciences Policy, Forum on Neuroscience and Nervous System Disorders, Forum on Drug Discovery, Development, and Translation (2024). Adult Attention-Deficit/Hyperactivity Disorder: Diagnosis, Treatment, and Implications for Drug Development: Proceedings of a Workshop.

[6] Gnanavel S., Sharma P., Kaushal P., Hussain S. (2019). Attention deficit hyperactivity disorder and comorbidity: A review of literature. World J. Clin. Cases.

[7] Pehlivanidis A., Papanikolaou K., Mantas V., Kalantzi E., Korobili K., Xenaki L.-A., Vassiliou G., Papageorgiou C. (2020). Lifetime co-occurring psychiatric disorders in newly diagnosed adults with attention deficit hyperactivity disorder (ADHD) or/and autism spectrum disorder (ASD). BMC Psychiatry.

[8] Schein J., Adler L.A., Childress A., Gagnon-Sanschagrin P., Davidson M., Kinkead F., Cloutier M., Guérin A., Lefebvre P. (2022). Economic burden of attention-deficit/hyperactivity disorder among adults in the United States: A societal perspective. J. Manag. Care Spéc. Pharm..

[9] Drechsler R., Brem S., Brandeis D., Grünblatt E., Berger G., Walitza S. (2020). Current concepts and treatments in children and adoles-cents. Neuropediatrics.

[10] Fuermaier A.B.M., Tucha L., Butzbach M., Weisbrod M., Aschenbrenner S., Tucha O. (2021). ADHD at the workplace: ADHD symptoms, diagnostic status, and work-related functioning. J. Neural Transm..

[11] Lopez P.L., Torrente F.M., Ciapponi A., Lischinsky A.G., Cetkovich-Bakmas M., Rojas J.I., Romano M., Manes F.F. (2018). Cognitive-behavioural interventions for attention deficit hyperactivity disorder (ADHD) in adults. Cochrane Database Syst. Rev..

[12] Tuan W.-J., Babinski D.E., Rabago D.P., Zgierska A.E. (2022). Treatment with stimulants and the risk of COVID-19 complications in adults with ADHD. Brain Res. Bull..

[13] Fredriksen M., Halmøy A., Faraone S.V., Haavik J. (2013). Long-term efficacy and safety of treatment with stimulants and atomoxetine in adult ADHD: A review of controlled and naturalistic studies. Eur. Neuropsychopharmacol..

[14] Susmarini D., Shin H., Choi S. (2024). Telehealth implementation for children with attention deficit hyperactivity disorder: A scoping review. Child Health Nurs. Res..

[15] Snoswell C., Chelberg G., De Guzman K., Haydon H., Thomas E., Caffery L., Smith A.C. (2023). The clinical effectiveness of telehealth: A systematic review of meta-analyses from 2010 to 2019. J. Telemed. Telecare.

[16] World Health Organization (2020). The Impact of COVID-19 on Mental, Neurological and Substance Use Services: Results of a Rapid Assessment.

[17] Stein M.A. (2022). Editorial Perspective: COVID-19, ADHD management and telehealth: Uncertain path. J. Child Psychol. Psychiatry.

[18] Mava Medical (2024). ADHD Telehealth Diagnosis and Treatment. https://mavamedical.com/adhd-telehealth-diagnosis-and-treatment/.

[19] Drug Enforcement Administration (2024). Proposed Adjustments to the Aggregate Production Quotas for Schedule I and II Controlled Substances. https://www.federalregister.gov/documents/2024/09/25/2024-21960/pro-posed-adjustments-to-the-aggregate-production-quotas-for-schedule-i-and-ii-controlled-substances.

[20] Nelson D., Inghels M., Kenny A., Skinner S., McCranor T., Wyatt S., Phull J., Nanyonjo A., Yusuff O., Gussy M. (2023). Mental health professionals and telehealth in a rural setting: A cross sectional survey. BMC Health Serv. Res..

[21] Chakrabarti S. (2015). Usefulness of telepsychiatry: A critical evaluation of videoconferencing-based approaches. World J. Psychiatry.

[22] Franciosi E.B., Tan A.J., Kassamali B., Leonard N., Zhou G., Krueger S., Rashighi M., LaChance A. (2021). The Impact of Telehealth Implementation on Underserved Populations and No-Show Rates by Medical Specialty During the COVID-19 Pandemic. Telemed. e-Health.

[23] Savage W.M., Saint-Hilaire S.A., Shah M., Lugo-Candelas C. (2024). Racial disparities in the diagnosis of disruptive behavior disorders: A U.S. national inpatient sample analysis. Front. Psychiatry.

[24] Berkman L.F., Kawachi I., Glymour M.M. (2014). Social Epidemiology.

[25] U.S. Department of Health and Human Services, Office of Disease Prevention and Health Promotion Social Determinants of Health. https://health.gov/healthypeople/objectives-and-data/social-determinants-health.

[26] Vakkalanka J.P., Gadag K., Lavin L., Ternes S., Healy H.S., Merchant K.A.S., Scott W., Wiggins W., Ward M.M., Mohr N.M. (2024). Telehealth Use and Health Equity for Mental Health and Substance Use Disorder During the COVID-19 Pandemic: A Systematic Review. Telemed. J. E-Health.

[27] Kino S., Hsu Y.-T., Shiba K., Chien Y.-S., Mita C., Kawachi I., Daoud A. (2021). A scoping review on the use of machine learning in research on social determinants of health: Trends and research prospects. SSM-Popul. Health.

[28] Athey S., Tibshirani J., Wager S. (2019). Generalized random forests. Ann. Stat..

[29] Hastie T. (2009). The Elements of Statistical Learning: Data Mining, Inference, and Prediction.

[30] Hutchings A., Hollywood J., Lamping D.L., Pease C.T., Chakravarty K., Silverman B., Choy E.H.S., Scott D.G., Hazleman B.L., Bourke B. (2007). Clinical outcomes, quality of life, and diagnostic uncertainty in the first year of polymyalgia rheumatica. Arthritis Rheum..

[31] Clare C.A. (2021). Telehealth and the digital divide as a social determinant of health during the COVID-19 pandemic. Netw. Model. Anal. Health Inform. Bioinform..

[32] Powell R.E., Henstenburg J.M., Cooper G., Hollander J.E., Rising K.L. (2017). Patient Perceptions of Telehealth Primary Care Video Visits. Ann. Fam. Med..

[33] Zhang D., Shi L., Han X., Li Y., Jalajel N.A., Patel S., Chen Z., Chen L., Wen M., Li H. (2021). Disparities in telehealth utilization during the COVID-19 pandemic: Findings from a nationally representative survey in the United States. J. Telemed. Telecare.

[34] Uscher-Pines L., Arora N., Jones M., Lee A., Sousa J.L., McCullough C.M., Lee S., Martineau M., Predmore Z., Whaley C.M. (2022). Experiences of Health Centers in Implementing Telehealth Visits for Underserved Patients During the COVID-19 Pandemic: Results from the Connected Care Accelerator Initiative. Rand Health Q..

[35] Lans A., Kanbier L.N., Bernstein D.N., Groot O.Q., Ogink P.T., Tobert D.G., Verlaan J., Schwab J.H. (2022). Social determinants of health in prognostic machine learning models for orthopaedic outcomes: A systematic review. J. Eval. Clin. Pract..

[36] Segar M.W., Hall J.L., Jhund P.S., Powell-Wiley T.M., Morris A.A., Kao D., Fonarow G.C., Hernandez R., Ibrahim N.E., Rutan C. (2022). Machine Learning–Based Models Incorporating Social Determinants of Health vs Traditional Models for Predicting In-Hospital Mortality in Patients With Heart Failure. JAMA Cardiol..

[37] Cengil A.B., Eksioglu B., Eksioglu S.D., Hayes C., Bogulski C., Ali M. (2026). Resource Use Patterns in US Telehealth Services: Machine Learning and Clustering Analysis Across 4 Specialties. JMIR Med. Inform..

[38] Chen S., Bergman D., Miller K., Kavanagh A., Frownfelter J., Showalter J. (2020). Using applied machine learning to predict healthcare utilization based on socioeconomic determinants of care. Am. J. Manag. Care.

[39] Chen T., Tachmazidis I., Batsakis S., Adamou M., Papadakis E., Antoniou G. (2023). Diagnosing attention-deficit hyperactivity disorder (ADHD) using artificial intelligence: A clinical study in the UK. Front. Psychiatry.

[40] Rohini B., Shoaib K., Yogish H. (2024). A review on machine learning approaches in diagnosis of ADHD based on big data. Big Data Computing.

[41] Center for Disease Control and Prevention (2025). Health Topics. https://www.cdc.gov/nchs/rss/rss-topics.html.

[42] Michelini G., Jurgiel J., Bakolis I., Cheung C.H.M., Asherson P., Loo S.K., Kuntsi J., Mohammad-Rezazadeh I. (2019). Atypical functional connectivity in adolescents and adults with persistent and remitted ADHD during a cognitive control task. Transl. Psychiatry.

[43] Agnew-Blais J.C., Polanczyk G.V., Danese A., Wertz J., Moffitt T.E., Arseneault L. (2018). Young adult mental health and functional outcomes among individuals with remitted, persistent and late-onset ADHD. Br. J. Psychiatry.

[44] Equator Network (2015). STROBE Checklist v4: Cross-Sectional Studies. https://www.equator-network.org/wp-content/uploads/2015/10/STROBE_checklist_v4_cross-sectional.pdf.

[45] Center for Disease Control and Prevention (2025). Questionnaire from National Center for Health Statistics. https://www.cdc.gov/nchs/data/rss/round2/questionnaire.pdf.

[46] Snell J., Swersky K., Zemel R. Prototypical Networks for Few-Shot Learning. Proceedings of the 31st International Conference on Neural Information Processing Systems (NeurIPS).

[47] Qin W. (2026). qwj0910/ADHD-Telehealth-SDOH-ML.

[48] Mathematica (2025). Barriers to Attention-Deficit/Hyperactivity Disorder Diagnosis in Adults: Findings from a Qualitative Study.

[49] Chung W., Jiang S.-F., Paksarian D., Nikolaidis A., Castellanos F.X., Merikangas K.R., Milham M.P. (2019). Trends in the Prevalence and Incidence of Attention-Deficit/Hyperactivity Disorder Among Adults and Children of Different Racial and Ethnic Groups. JAMA Netw. Open.

[50] Fairman K.A., Peckham A.M., Sclar D.A. (2017). Diagnosis and Treatment of ADHD in the United States: Update by Gender and Race. J. Atten. Disord..

[51] Kessler R.C., Adler L., Barkley R., Biederman J., Conners C.K., Demler O., Faraone S.V., Greenhill L.L., Howes M.J., Secnik K. (2006). The prevalence and correlates of adult ADHD in the United States: Results from the National Comorbidity Survey Replication. Am. J. Psychiatry.

[52] Children and Adults with Attention-Deficit/Hyperactivity Disorder (CHADD) (2024). General Prevalence of ADHD in Adults. https://chadd.org/about-adhd/general-prevalence-adults/.

[53] Landes S.D., London A.S. (2018). Self-Reported ADHD and Adult Health in the United States. J. Atten. Disord..

[54] Barkley R.A., Murphy K.R., Fischer M. (2008). ADHD in Adults: What the Science Says.

[55] Biederman J., Faraone S.V. (2006). The effects of attention-deficit/hyperactivity disorder on employment and household income. Medscape Gen. Med..

[56] Das D., Cherbuin N., Butterworth P., Anstey K.J., Easteal S. (2012). A Population-Based Study of Attention Deficit/Hyperactivity Disorder Symptoms and Associated Impairment in Middle-Aged Adults. PLoS ONE.

[57] de Zwaan M., Gruß B., Müller A., Graap H., Martin A., Glaesmer H., Hilbert A., Philipsen A. (2012). The estimated prevalence and correlates of adult ADHD in a German community sample. Eur. Arch. Psychiatry Clin. Neurosci..

[58] Lensing M.B., Zeiner P., Sandvik L., Opjordsmoen S. (2013). Quality of Life in Adults Aged 50+ With ADHD. J. Atten. Disord..

[59] Spencer T.J., Biederman J., Mick E. (2007). Attention-Deficit/Hyperactivity Disorder: Diagnosis, Lifespan, Comorbidities, and Neurobiology. J. Pediatr. Psychol..

[60] Michaëlsson M., Yuan S., Melhus H., Baron J.A., Byberg L., Larsson S.C., Michaëlsson K. (2022). The impact and causal directions for the associations between diagnosis of ADHD, socioeconomic status, and intelligence by use of a bi-directional two-sample Mendelian randomization design. BMC Med..

[61] RTTNews Staff Writer (2024). Over 15 Mln US Adults Have ADHD, Says New CDC Report. https://www.rttnews.com/3481216/over-15-mln-us-adults-have-adhd-says-new-cdc-report.aspx.

[62] Patil M., Konda S., Ganti L. (2024). Assessing ADHD prevalence and comorbidities in the United States: Insights from the Substance Abuse and Mental Health Services (SAMHSA) data. Glob. Ment. Health.

[63] Hu X., Graetz I., Marchak J., Mertens A., Ji X., Cummings J. (2025). Racial and Ethnic Disparities in Telemental Health Use Among Publicly Insured Children. Am. J. Manag. Care.

[64] Faraone S.V., Larsson H. (2019). Genetics of attention deficit hyperactivity disorder. Mol. Psychiatry..

