# Supplementary material for: Exploring the Association Between Social Determinants of Health and Telehealth Utilization for Attention-Deficit/Hyperactivity Disorder Among Adults Using Machine Learning: A Cross-Sectional Study
Source: Healthcare (Basel). 2026 Aug 25;14(17):2709. doi: 10.3390/healthcare14172709 (PMC13564688; doi:10.3390/healthcare14172709)
Supplement: Supplementary file 1 [file healthcare-14-02709-s001.zip › healthcare-4397026-supplementary.pdf]

Table S1: The Distributions for Social Determinants of Health (SDOH) by Attention Deficit Hyperactivity Disorder (ADHD)-Status Group

Variables	Category	Whole Pop (n)	Whole Pop (%)	Never ADHD (n)	Never ADHD (%)	Never 95% CI	Never p-value	Had ADHD (n)	Had ADHD (%)	Remit 95% CI	Remit p-value	Current ADHD (n)	Current ADHD (%)	Current 95% CI	Current p-value
Age							0.0038905				3.1471389e-12*				4.9673e-46*
	18-24 years	358	5.15%	294	4.6%	[0.041, 0.0513]		8	6.72%	[0.0874, 0.1404]		55	12.82%	[0.0966, 0.1598]	
	25-44 years	2229	32.04%	1901	29.8%	[0.2871, 0.3096]		74	62.18%	[0.5302, 0.6127]		240	55.94%	[0.5125, 0.6064]	
	45-59 years	1616	23.23%	1497	23.5%	[0.2245, 0.2453]		21	17.65%	[0.1707, 0.2379]		90	20.98%	[0.1713, 0.2483]	
	60-70 years	2753	39.58%	2680	42.1%	[0.4085, 0.4327]		16	13.45%	[0.0842, 0.1364]		44	10.26%	[0.0739, 0.1313]	
Household Size							0.7765991				0.1572638				2.101996e-07*
	1	1435	20.37%	1324	20.6%	[0.1978, 0.2177]		21	16.94%	[0.1555, 0.2206]		81	18.24%	[0.1518, 0.2258]	
	2	2661	37.77%	2495	38.7%	[0.3796, 0.4035]		39	31.45%	[0.2498, 0.3252]		118	26.58%	[0.2328, 0.3173]	
	3	1155	16.39%	1034	16.1%	[0.1532, 0.1713]		21	16.94%	[0.1775, 0.2456]		96	21.62%	[0.1843, 0.2632]	
	4	913	12.96%	820	12.7%	[0.1205, 0.1369]		20	16.13%	[0.1286, 0.1896]		67	15.09%	[0.1218, 0.1905]	
	5	478	6.78%	426	6.6%	[0.0607, 0.073]		10	8.06%	[0.0602, 0.1062]		36	8.11%	[0.0577, 0.1102]	
	6+	404	5.73%	342	5.3%	[0.0481, 0.0592]		13	10.48%	[0.081, 0.1324]		46	10.36%	[0.0779, 0.1365]	
Gender							0.5754899				0.0660337				0.02278184*
	Male	3250	46.13%	2939	45.6%	[0.449, 0.4735]		68	54.84%	[0.5046, 0.5876]		230	51.80%	[0.4889, 0.5833]	
	Female	3796	53.87%	3502	54.4%	[0.5374, 0.5618]		56	45.16%	[0.4484, 0.5317]		214	48.20%	[0.4515, 0.5461]	
Education Level							0.7645825				0.2309947				0.01176904*
	High school or less	2011	28.54%	1816	28.2%	[0.2739, 0.2961]		42	33.87%	[0.3022, 0.3813]		146	32.88%	[0.2955, 0.3852]	
	Some college	2257	32.03%	2046	31.8%	[0.3096, 0.3326]		42	33.87%	[0.3181, 0.398]		154	34.68%	[0.3136, 0.4044]	
	Bachelor's or higher	2778	39.43%	2579	40.0%	[0.3927, 0.4168]		40	32.26%	[0.297, 0.3757]		144	32.43%	[0.291, 0.3804]	
Household Income							0.8984700				0.1529941				0.6926213
	$0-39,999	282	29.47%	237	28.5%			10	43.48%			32	33.68%		
	$40,000-84,999	226	23.62%	198	23.8%	[0.0268, 0.0353]		5	21.74%	[0.0294, 0.0647]		21	22.11%	[0.0285, 0.0694]	
	$85,000+	449	46.92%	397	47.7%			8	34.78%			42	44.21%		
Race/Ethnicity							0.9690948				0.037505				0.03347356*
	White	4702	67.26%	4308	67.4%	[0.6646, 0.6876]		69	57.98%	[0.6373, 0.7153]		301	68.41%	[0.6583, 0.7449]	
	Black	728	10.41%	673	10.5%	[0.0981, 0.1132]		19	15.97%	[0.0665, 0.1143]		30	6.82%	[0.0458, 0.0941]	
	Other	595	8.51%	544	8.5%	[0.0785, 0.0922]		16	13.45%	[0.0665, 0.1143]		34	7.73%	[0.0537, 0.1048]	
	Hispanic	966	13.82%	865	13.5%	[0.1273, 0.1442]		20	16.81%	[0.1404, 0.2032]		75	17.05%	[0.1389, 0.2108]	
Poverty Ratio							0.9156738				0.0075147				1.56735e-07*
	<0.5	331	4.70%	272	4.2%	[0.0377, 0.0477]		11	8.87%	[0.0761, 0.1264]		44	9.91%	[0.0739, 0.1313]	
	0.50-1.00	507	7.20%	438	6.8%	[0.0625, 0.075]		11	8.87%	[0.0923, 0.1464]		55	12.39%	[0.0966, 0.1598]	
	1.00-1.50	480	6.81%	429	6.7%	[0.0612, 0.0735]		15	12.10%	[0.0633, 0.1103]		33	7.43%	[0.0517, 0.1021]	
	1.50-2.00	750	10.64%	688	10.7%	[0.1004, 0.1156]		15	12.10%	[0.0777, 0.1284]		41	9.23%	[0.0677, 0.1234]	
	2.00-2.50	474	6.73%	431	6.7%	[0.0615, 0.0738]		9	7.26%	[0.0507, 0.0939]		31	6.98%	[0.0478, 0.0968]	
	2.50-3.00	493	7.00%	450	7.0%	[0.0643, 0.0769]		11	8.87%	[0.0554, 0.1001]		32	7.21%	[0.0497, 0.0995]	
	3.00-3.50	662	9.40%	614	9.5%	[0.0891, 0.1036]		13	10.48%	[0.0586, 0.1042]		31	6.98%	[0.0478, 0.0968]	
	3.50-4.00	329	4.67%	300	4.7%	[0.0419, 0.0523]		8	6.45%	[0.0339, 0.071]		20	4.50%	[0.0267, 0.0666]	
	4.00+	3020	42.86%	2819	43.8%	[0.4302, 0.4546]		31	25.00%	[0.3022, 0.3813]		157	35.36%	[0.3204, 0.4116]	
Employment Status							0.1555338				0.0005817*				8.899652e-22*
	Working	4241	60.19%	3829	59.4%	[0.5889, 0.6129]		82	66.13%	[0.6729, 0.7485]		307	69.14%	[0.6729, 0.7583]	
	Unable to work	453	6.43%	383	5.9%	[0.0543, 0.0659]		14	11.29%	[0.0972, 0.1523]		55	12.39%	[0.0966, 0.1598]	
	Retired	1631	23.15%	1590	24.7%	[0.2389, 0.2602]		11	8.87%	[0.0369, 0.0752]		19	4.28%	[0.0248, 0.0638]	
	Other	721	10.23%	639	9.9%	[0.0929, 0.1077]		17	13.71%	[0.1153, 0.174]		63	14.19%	[0.1134, 0.1803]	
Marital Status							0.0779935				0.0020201				1.081287e-20*
	Married	3784	53.70%	3551	55.1%	[0.5451, 0.5695]		54	43.55%	[0.3553, 0.4368]		163	36.71%	[0.334, 0.4259]	
	Divorced/Separated	1038	14.73%	943	14.6%	[0.1393, 0.1567]		19	15.32%	[0.1236, 0.1838]		65	14.64%	[0.1176, 0.1854]	
	Widowed	375	5.32%	365	5.7%	[0.0516, 0.063]		1	0.81%	[0.0045, 0.0244]		7	1.58%	[0.0043, 0.0283]	
	Never married	1849	26.24%	1582	24.6%	[0.2377, 0.2589]		50	40.32%	[0.4304, 0.5136]		209	47.07%	[0.4399, 0.5345]	
Census Region							0.9614562				0.4435814				0.3252516
	Northeast	1081	15.34%	1000	15.5%	[0.148, 0.1659]		16	12.90%	[0.1104, 0.1681]		60	13.51%	[0.107, 0.1727]	
	Midwest	1672	23.73%	1515	23.5%	[0.2273, 0.2482]		30	24.19%	[0.2307, 0.3045]		117	26.35%	[0.2306, 0.3149]	
	South	2516	35.71%	2284	35.5%	[0.3467, 0.3702]		52	41.94%	[0.357, 0.4386]		166	37.39%	[0.3409, 0.433]	
	West	1777	25.22%	1642	25.5%	[0.247, 0.2684]		26	20.97%	[0.1963, 0.2666]		101	22.75%	[0.1953, 0.2756]	
Home Ownership							0.1137657				2.7074e-06*				7.25009e-15*
	Owned	4988	70.79%	4664	72.4%	[0.7211, 0.7428]		62	50.00%	[0.4992, 0.5822]		234	52.70%	[0.4983, 0.5926]	
	Rented	1922	27.28%	1658	25.7%	[0.2494, 0.271]		57	45.97%	[0.4232, 0.5063]		198	44.59%	[0.4144, 0.5087]	
	No rent	136	1.93%	119	1.8%	[0.0154, 0.022]		5	4.03%	[0.0164, 0.0451]		12	2.70%	[0.0124, 0.0436]	
Metropolitan Status							0.8821201				0.7641420				0.2856546
	Metro	6041	85.74%	5529	85.8%	[0.8594, 0.876]		108	87.10%	[0.8497, 0.9044]		372	83.78%	[0.835, 0.8993]	
	Non-metro	1005	14.26%	912	14.2%	[0.1345, 0.1517]		16	12.90%	[0.1286, 0.1896]		72	16.22%	[0.1325, 0.2032]	
Health Insurance							0.5576923				0.1142809				2.646002e-05*
	Insured	5033	71.43%	4653	72.2%	[0.7193, 0.7411]		78	62.90%	[0.6038, 0.6837]		274	61.71%	[0.5932, 0.6842]	
	Uninsured	360	5.11%	326	5.1%	[0.0458, 0.0566]		8	6.45%	[0.0384, 0.0773]		24	5.41%	[0.0342, 0.0777]	
	No response	1653	23.46%	1462	22.7%			38	30.65%			146	32.88%		
Difficulty Paying Bills							0.0023022				8.121982e-06*				8.54487e-35*
	No	5896	84.36%	5512	86.2%			85	69.11%			270	61.50%		
	Yes	1093	15.64%	879	13.8%			38	30.89%			169	38.50%		
Worry About Medical Bills							0.0663050				0.023458				1.31163e-22*
	Very worried	970	13.82%	808	12.6%			24	19.83%			132	30.00%		
	Somewhat worried	2736	38.98%	2488	38.7%			54	44.63%			177	40.23%		
	Not at all worried	3313	47.20%	3125	48.7%			43	35.54%			131	29.77%		
Housing Cost Burden							0.0011946				3.132245e-05*				8.032134e-39*
	No	6107	87.41%	5705	89.2%			89	74.17%			285	65.07%		
	Yes	880	12.59%	689	10.8%			31	25.83%			153	34.93%		
Transportation Barriers							0.0004280				5.71787e-11*				1.4176494e-38*
	No lack	6393	91.38%	5955	93.0%			91	74.59%			315	72.25%		
	Lack of	603	8.62%	446	7.0%			32	26.23%			121	27.75%		
Usual Place for Care							0.884367				0.013735*				0.168132
	Single usual place	4119	58.68%	3790	59.1%			63	51.64%			240	54.18%		
	No place	582	8.29%	521	8.1%			19	15.57%			39	8.80%		
	More than one	2319	33.03%	2107	32.8%			40	32.79%			164	37.02%		
Type of Usual Care							0.892697				0.000366313*				0.002865*
	Do not go to one	89	1.38%	83	1.4%			2	1.94%			4	1.00%		
	Doctor's office/center	5333	82.97%	4924	83.6%			73	70.87%			307	76.37%		

Note: Pop: population

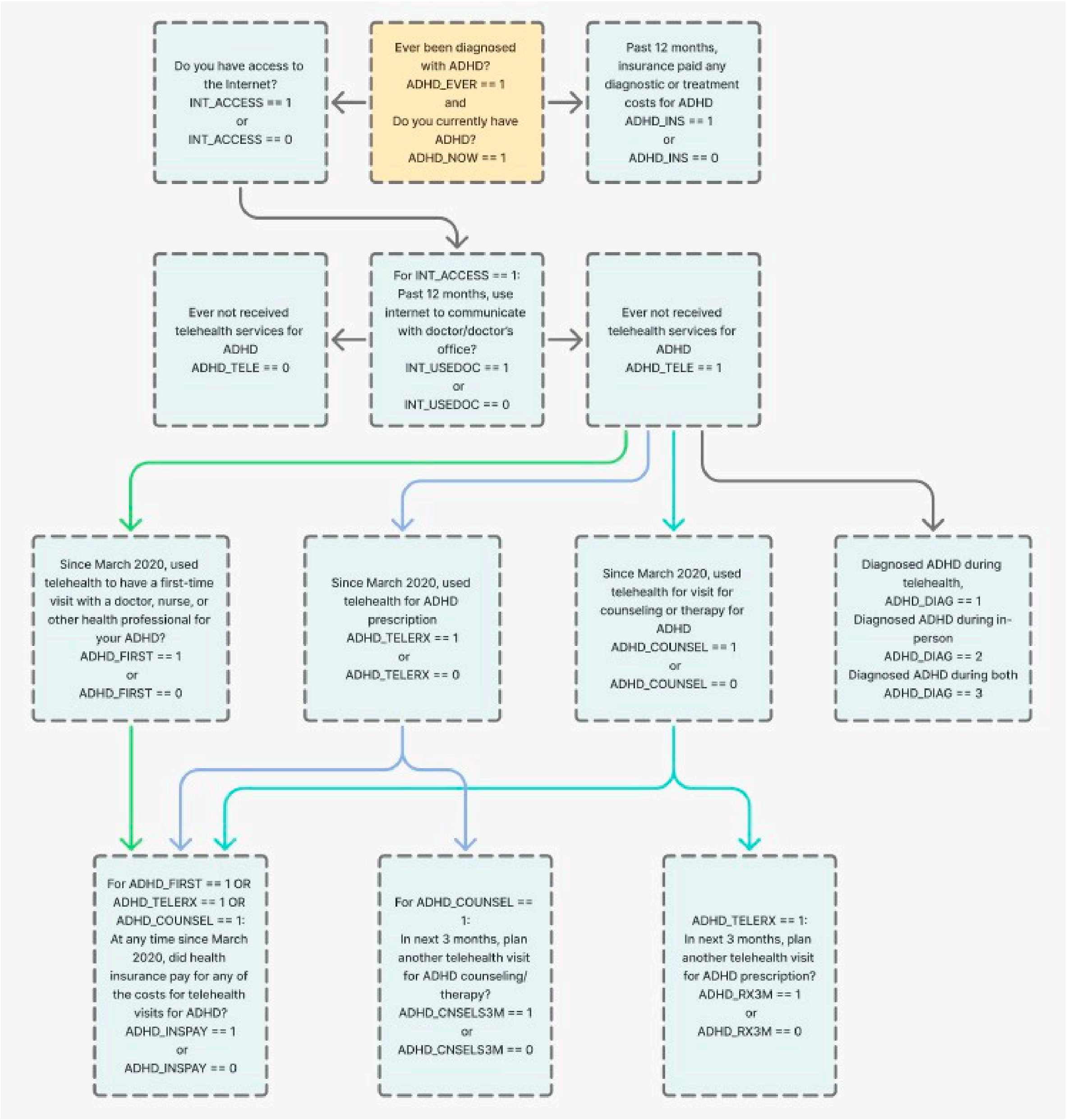

Figure S1: Utilization of Telehealth Services for Attention Deficit Hyperactivity Disorder (ADHD) Patients

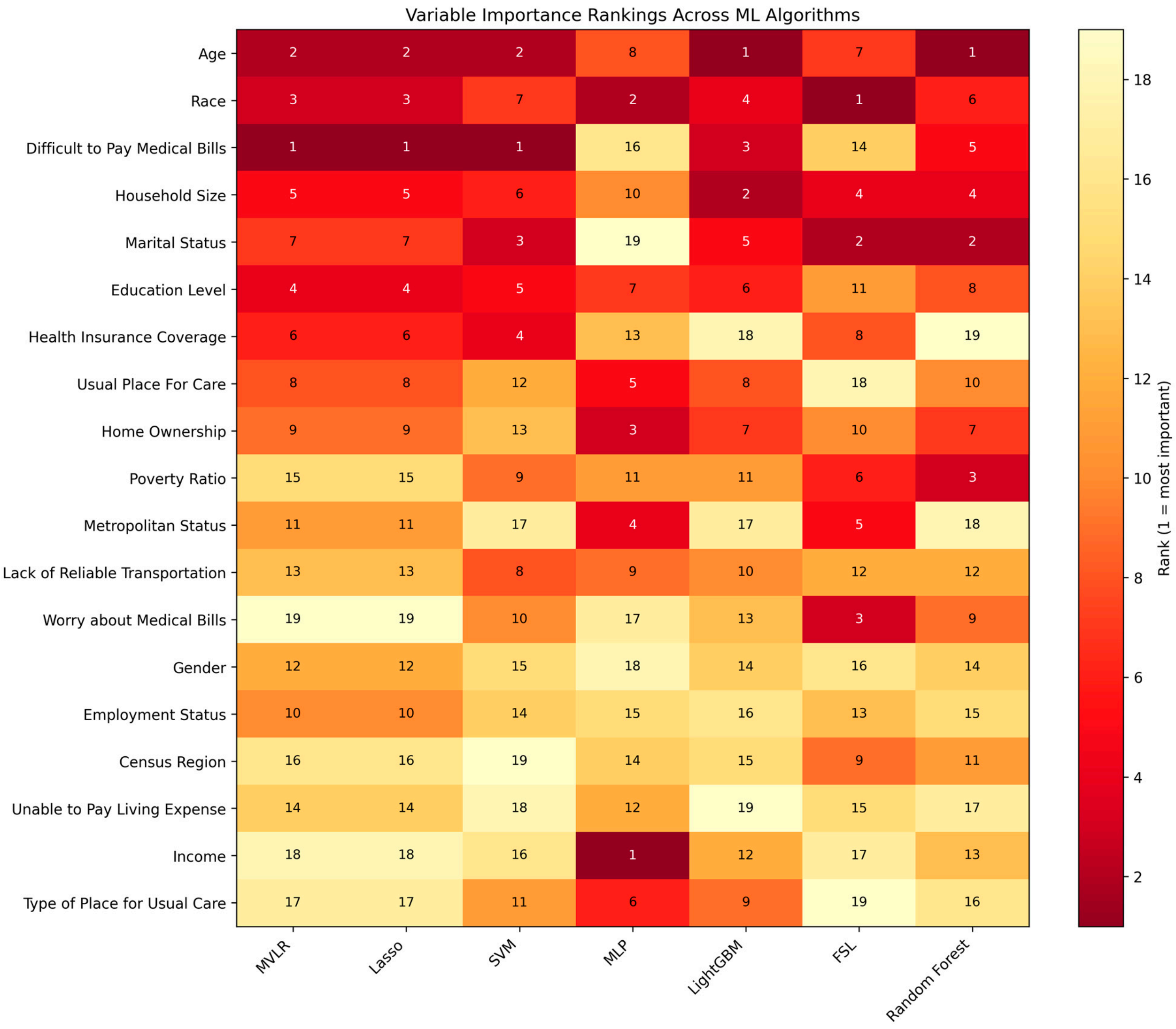

Figure S2: Variable Importance Ranking Across Machine Learning (ML) Algorithms

Table S2:Hyperparameters details

ML Algorithm	Hyperparameters
Random Fores	n_estimators = 500; max_depth = 8; min_samples_split = 4; min_samples_leaf = 8; max_features = 0.4; class_weight = balanced
SVM	kernel = linear; C = 0.12; gamma = auto; class_weight = balanced
Elastic Net	C = 0.37; L1_ratio = 0.9; class_weight = balanced
Lasso	C = 1.0; class_weight = balanced
MLP	hidden_layers = (64, 64); learning_rate_init = 0.003; learning_rate = adaptive; batch_size = 128; alpha = 1e-6; activation = tanh
LightGBM	n_estimators = 400; max_depth = 10; learning_rate = 0.01; num_leaves = 31; min_child_samples = 40; subsample = 0.8; colsample_bytree = 0.7; class_weight = balanced
FSL	hidden_dim = 64; dropout = 0.2; learning_rate = 0.0003; batch_size = 32; epochs = 100
